# Paleoproteomic profiling of organic residues on prehistoric pottery from Malta

**DOI:** 10.1007/s00726-021-02946-4

**Published:** 2021-02-13

**Authors:** Davide Tanasi, Annamaria Cucina, Vincenzo Cunsolo, Rosaria Saletti, Antonella Di Francesco, Enrico Greco, Salvatore Foti

**Affiliations:** 1 Department of History, University of South Florida, SOC107 4202 E. Fowler Ave, Tampa, FL 33620 USA; 2 Laboratory of Organic Mass Spectrometry, Department of Chemical Sciences, University of Catania, Viale A. Doria 6, 95125 Catania, Italy; 3 Aix-Marseille Université, Institut de Chimie Radicalaire, Service 512, Avenue Escadrille Normandie Niemen, 13013 Marseille, France

**Keywords:** Paleoproteomics, Cereal proteins, High-resolution mass spectrometry, Orbitrap fusion tribrid, Deamidation

## Abstract

Mass spectrometry-based approaches have been successfully applied for identifying ancient proteins in bones and other tissues. On the contrary, there are relatively few examples of the successful recovery and identification of archeological protein residues from ceramic artifacts; this is because ceramics contain much lower levels of proteins which are extensively degraded by diagenetic effects. In this paper, we report the results of the characterization of proteins extracted from pottery of the Maltese site of Baħrija, the guide-site for the Baħrija period (half of 9th–second half of eighth century BCE), recently identified as the final part of the Borġ in-Nadur culture. Proteomic data here reported confirm that one of the major issue of these kind of studies is represented by contamination of animal and human agents that may complicate endogenous protein identification and authentication. The samples tested included a small group of ceramic forms, namely three tableware and six coarse ware thought to have been used in food preparation and/or storage. In this context, the limited availability of paleobotanical and archeozoological analyses may be compensated by the outcomes of the first proteomics profiling which, even if obtained on a limited selection of vessels, revealed the centrality of wheat in the diet of the ancient community of Baħrija. The data have been deposited to the ProteomeXchange with identifier < PXD022848 > .

**Supplementary Information:**

The online version contains supplementary material available at (10.1007/s00726-021-02946-4).

## Introduction

Over the past three decades, studies of ancient biomolecules, in particular ancient DNA (aDNA), proteins, and lipids, allowed to shed light on  the biological past, improving our understanding of evolutionary history (Cappellini et al., 2018). Particularly, the interest in the study of paleo-diets has grown considerably in tandem with the use of technologies and analytical tools that have made it possible to identify the organic residues more precisely. The use of multiple techniques to validate scientific results has been repeatedly used to identify animal products (Mottram et al. 1999; Spangenberg et al. 2006; Spiteri et al. 2020), milk (Dudd et al. 1998; Evershed et al. 2002) and vegetable lipids (Colombini et al. 2005), fish and shellfish (Hansel et al. 2004) or to check the presence of fermented drinks such as grape wine (Cavalieri et al. 2003; Guasch-Jané et al. 2004), beer (Borek et al. 2007), or other beverages (McGovern et al. 2013). Studies on plant microremains and aDNA trapped in ancient human dental calculus have also been carried out, providing new insights about past human diet and health (Weyrich et al. 2015; Leonard et al. 2015; Søe et al. 2018; Gismondi et al. 2020). On this respect, dental calculus, a mineralized form of dental plaque, represents a long-term reservoir of dietary biomolecules and microfossils, offering a unique opportunity to access primary evidence of ancient diets at an individual level (Hendy et al. 2018a; Velsko et al. 2017; Warinner et al. 2014, 2015).

Proteomics has been widely applied to detect and identify proteins on paleontological and archaeological artefacts. The first research on such interdisciplinary approach dates to early 2000 (Ostrom et al. 2000; Tokarski et al. 2003) and, since then, investigations on ancient samples are steadily increasing (Cappellini et al. 2014a; Dallongeville et al. 2016). Such growing popularity is undoubtedly determined by the significant technological development, in terms of performance and versatility of the MS instrumentation which nowadays represents an indispensable tool in paleoproteomics (Cleland et al. 2018). This has facilitated the phylogenetic identification of extant and extinct species (Cappellini et al. 2014b; Welker et al., 2015), studies of diagenetic and in vivo protein post-translational modifications (PTMs) (Cleland et al. 2015; Mikšík et al. 2016), the characterization of past human diseases (Hendy et al. 2016; D’Amato et al. 2018), and the reconstruction of the human diet (Warinner et al. 2014; Yang et al 2014; Shevchenko et al. 2014; Xie et al. 2016; Greco et al. 2018; Charlton et al. 2019). In comparison with the apparent widespread success of mass spectrometry approaches for identifying ancient proteins in bones and other tissues (Dallongeville et al. 2016), there are relatively few examples of the successful recovery and identification of archeological protein residues from ceramic artifacts (Solazzo et al. 2008; Dallongeville et al. 2011; Buckley et al. 2013; Salque et al. 2013; Shevchenko et al. 2018; Hendy et al. 2018b). Mostly because ceramics contain a much lower amount of proteinaceous material. The above mentioned studies also highlight how challenges routinely come from the heterogenous and unusual physical state of ancient samples and from the high level of degradation (Vinciguerra et al. 2016; Barker et al. 2018). The other major issue is represented by contamination of animal and human agents which could have occurred at pre-depositional (the contamination that a vessel sustains during its ‘life’) depositional (the contamination it sustains when it becomes part of an archeological context) and post-depositional stage (the contamination it sustains from the time it is unearthed to that when its sample reaches a proteomic laboratory) (Skibo et al. 2015). Concerning contamination, the challenge of discerning contaminant proteins from endogenous ancient molecules becomes particularly problematic. Authentication and validation criteria are needed to discriminate ancient endogenous proteins from contaminants. Deamidation process of asparagine and mainly of glutamine residues is the proposed marker of age that has recently been used in many archeological and paleontological studies (Robinson et al. 2001; Robinson 2002; Wilson et al. 2012; Van Doorn et al. 2012). With  this respect, taking into account the complexity and the peculiarity of archeological samples and also their inestimable value, during the last decade conventional proteomics protocols have been optimized and a series of precautions, from sample selection to data interpretation, have been successfully set, especially for bone remains (Hendy et al. 2018c). On the other hand, the studies on protein residues from ceramics highlighted that diagenesis deeply affects the protein sequences. Diagenesis, including a complex network of reactions such as chemical degradation and molecular breakdown driven by environmental factors during burial and storage, results in protein modifications beyond those produced in vivo and introduces a new challenge for protein identification and authentication (Cleland et al. 2020). Some substrates (e.g., bone, dental calculus, and eggshell) may harbor a better potential for preserving endogenous proteins than other ones. On the contrary, on ancient pottery, the microbial attack of the diagenetic action are more extensive because of the poorer screen effect of ceramics compared to the hydroxyapatite cage that protects bone proteins. However, it should be noted that calcified deposits lining the inner surfaces of archeological vessels may facilitate long-term archeological preservation of proteins and peptides (Hendy et al. 2018b). Detection of surviving proteins is also hampered by their hydrophilic properties (compared to lipids, for example), solubility in water, and the matrix effect of humics. Consequently, the identification of proteins in pottery is extremely challenging compared to other archaeological samples. It should be highlighted that another challenge, for which it does not exist any definitive solution, is related to the reuse of ancient pottery. Indeed, pottery was often subject to reuse for different food preparation purposes, leaving behind a complex and stratified combination of protein traces. However, information derived from an archeological context, a typological study of the pottery, analysis of written and iconographic sources, and ethno-archeological research allow us to significantly narrow down the use or number of uses done for certain vessels (Skibo et al. 2012). Altogether, these aspects highlight the challenges of paleoproteomic studies, such as the characterization of ancient proteins absorbed by archeological pottery. Continuous efforts are aimed to improve experimental and bioinformatic strategies for characterizing protein degradation and contamination, as well as for protein validation and authentication.

Within this research line, this paper reports the results of the first-ever characterization of protein extracted from prehistoric pottery of the Maltese Bronze/Iron Age site of Baħrija, via a shotgun MS-based approach.

## Materials and methods

### Case study: the iron age settlement at Qlejgħa tal-Baħrija (Malta)

**Fig. 1:**
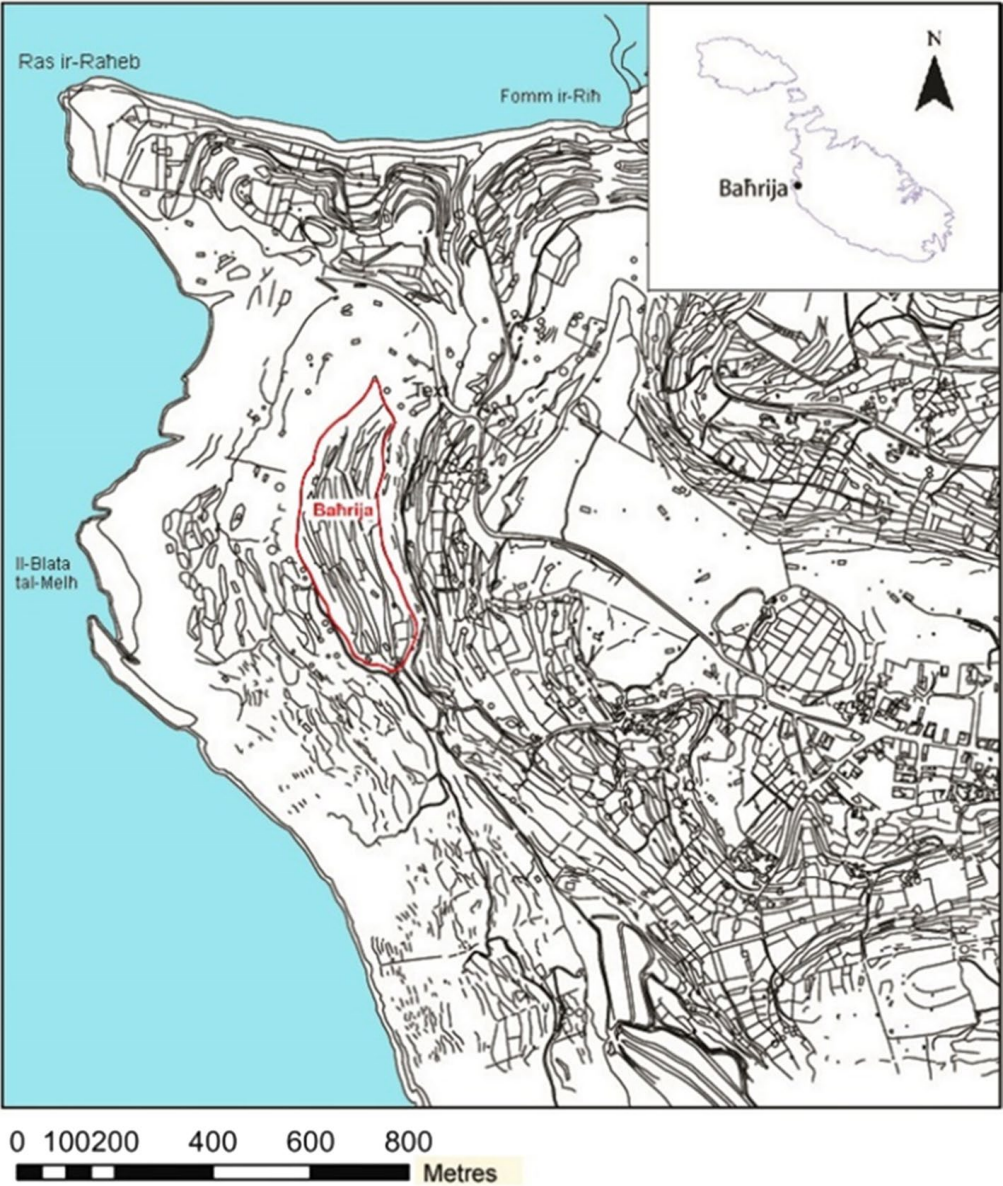
Contour Map of il-Qlejgħa tal-Baħrija area, NW Malta (after Cardona and Zammit 2015)

**Fig. 2:**
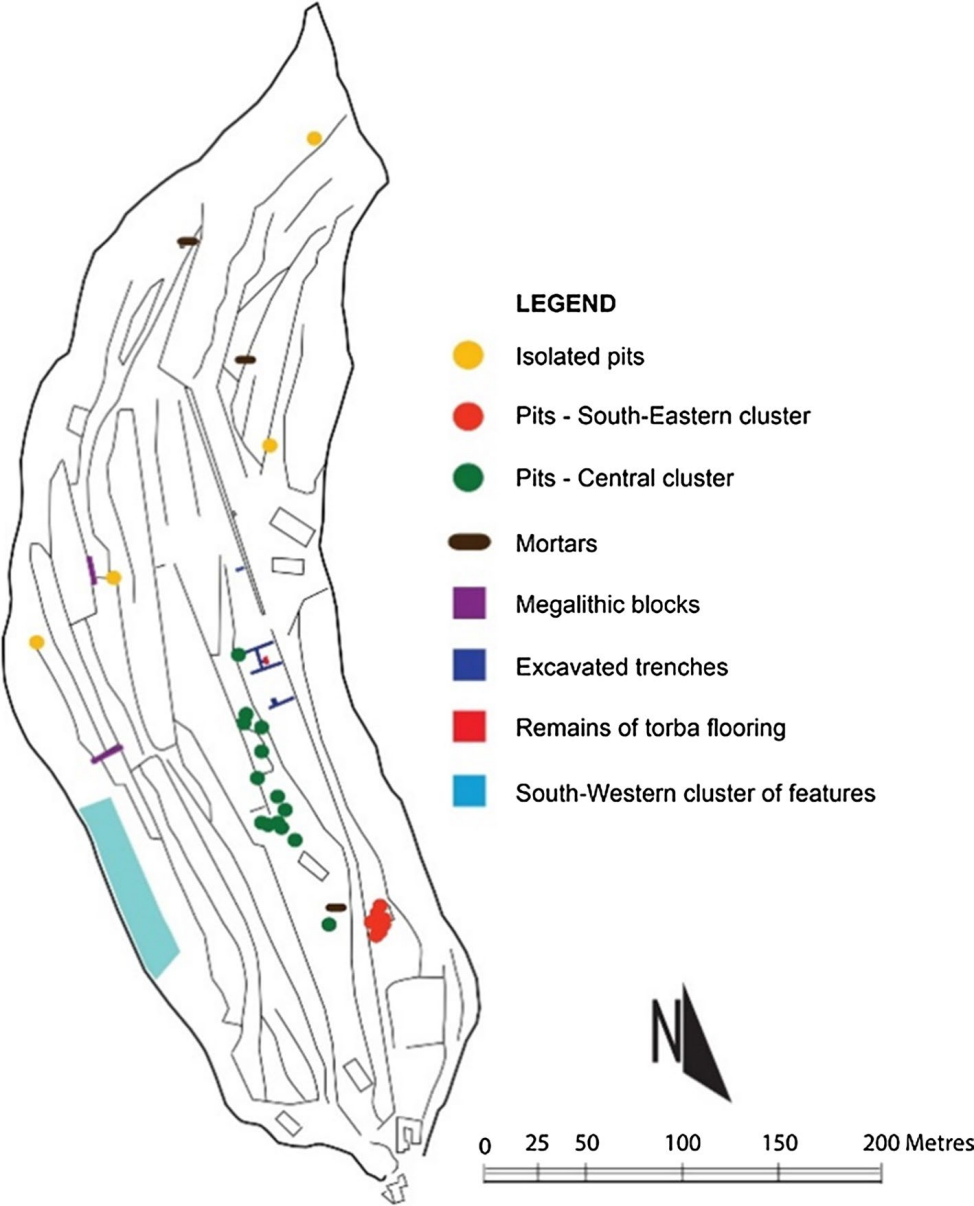
General site plan of il-Qlejgħa tal-Baħrija showing all the known archeological features (Cardona and Zammit 2015)

Il-Qlejgħa tal-Baħrija is an Upper Coralline Limestone plateau on the west coast of Malta. Flanked on the eastern side by some of the most fertile lands in the area and offering the strategic advantage and the natural defense of the high ground, the Qlejgħa tal-Baħrija hilltop attracted the attention of human activity in prehistory when a settlement was established on it (Fig. 1). The first archeological exploration of the site was carried out by T.E. Peet in June 1909 (Peet 1910) with a series of long and narrow trenches which intercepted, at about 30–50 cm of depth, a gray layer of soil characterized by abundant pottery and bones (layer 5) and related with at least two structures likely to be interpreted as wattle and daub huts (in trench M and trench G) (Cardona 2020). Among the materials from layer 5, the vast majority of the vessels were tableware (cups, bowl, jugs) and coarse ware (storage jars) of medium–large size (50–100 cm in height), mostly decorated with incisions and impressions typical of the Baħrija style which emerges in the final stage of the Maltese Bronze Age. Recent studies have characterized such stage as a ‘Bahrija period’, assignable to mid-9th to mid-eighth century BCE, corresponding to the latest development of the Borġ in-Nadur culture (Tanasi 2020). In 1959, further excavations conducted by D.H. Trump, not far from the area previously investigated, uncovered several other significant features on the plateau and confirmed the evidence of a well-organized settlement (Fig. 2) (Trump 1961). After 1959, no further excavations were ever carried out in the site. However, the publication of an overall reappraisal of the legacy excavation data (Tanasi and Cardona 2020) has now shown evidence of a first Maltese proto-urban society, able to establish new connections with Sicilian and Aegean communities and exploiting the strategic geography of Malta, at the center of the Mediterranean basin, to influence major historical events before the Greek and Phoenician colonizations BCE (Bonanno 2017).

### Chemicals

The chemicals employed during the course of the analysis were of the highest purity commercially available and used without further purification. Tris–HCl was purchased from Carlo Erba (Milan, Italy); formic acid (FA), ammonium bicarbonate, dithiothreitol (DTT), iodoacetamide (IAA) from Aldrich (St. Louis, Missouri, USA), Trifluoracetic acid (TFA) from Riedel-de Haën; modified porcine trypsin and sodium dodecyl sulfate (SDS) from Promega (Madison, WI, USA); water and acetonitrile (ACN) (OPTIMA® LC/MS grade) for LC/MS analyses from Fisher Scientific (Milan, Italy). All the chemicals listed above were exclusively employed for the present study.

### Sample collection

In consideration of the historical importance of the site of Qlejgħa tal-Baħrija, it becomes crucial to determine whether at the site there was a practice of cereal storage, which in the contemporaneous Sicilian society was an indication of advanced food redistribution policies (Albanese Procelli 2003). To investigate the foodways of the prehistoric community of Qlejgħa tal-Baħrija, in absence of paleobotanical and archeozoological data, and to determine the eventual use for cereal storage of the numerous large storage jars found on site, it was decided to sample specific shapes from the tableware and coarse ware repertoire to perform chemical analyses and explore the possible presence of organic residues. To this extent, in summer 2017, nine samples (three of tableware and six of coarse ware) were extracted from vessels related to the Late Borġ in-Nadur style and Baħrija style from the Peet excavation (Table 1).

**Table 1:**
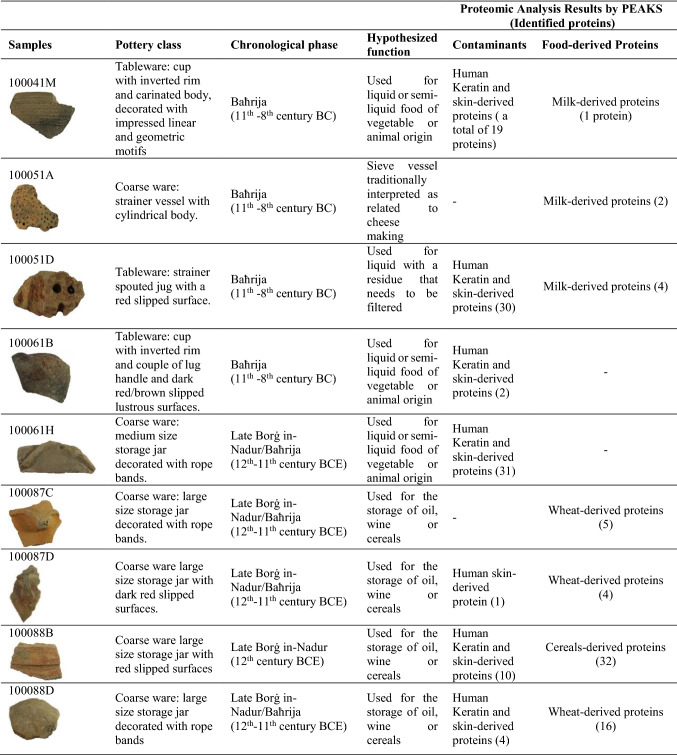
List of pottery remains analyzed in the present work

The list of food-derived proteins identified by the MS-based proteomic analysis is reported in the Table 2. The complete set of proteins, including potential contaminants, is reported in the Supplementary Table S1

**Table 2:** List of the food-derived proteins identified by PEAKS search in the archeological samples here investigated

Sample	Proteins	UniProt ID	Taxa	InChorus Score (%)	Coverage (%)	Peptides	Unique	
**100041 M**	Alpha-S1-casein	P02662	*Bos taurus*	97.97	10	3	3	
**100051A**								
	Beta-lactoglobulin	P02754	*Bos taurus*	83.68	8	2	2	Multispecies identification: Bubalus bubalis
	Beta-casein	P02666	*Bos taurus*	76.82	12	2	2	Multispecies identification: Bubalus bubalis
**100051D**	Kappa-casein	P02668	*Bos taurus*	98.24	20	3	3	
	Alpha-S1-casein	P02662	*Bos taurus*	97.35	21	6	6	
	Alpha-S2-casein	P02663	*Bos taurus*	83.61	15	2	2	Multispecies identification: Ovis aries
	Beta-casein	P02666	*Bos taurus*	79.64	12	3	3	Multispecies identification: Bubalus bubalis
**100061B**	No Id							
**100061H**	No Id							
**100087C**	Alpha-amylase/trypsin inhibitor CM3	P17314	*Triticum aestivum*	98.35	25	2	2	
	Purothionin A-1	P01543	*Triticum aestivum*	98.29	26	4	1	
	Non-specific lipid-transfer protein (Fragment)	P24296	*Triticum aestivum*	98.25	45	3	3	
	Alpha-1-purothionin (Fragment)	P01544	*Triticum aestivum*	98.24	27	3	2	
	Puroindoline-B	Q10464	*Triticum aestivum*	75.95	18	2	2	
**100087D**	Alpha-amylase/trypsin inhibitor CM3	P17314	*Triticum aestivum*	98.41	40	3	3	
	Alpha-amylase/trypsin inhibitor CM16	P16159	*Triticum aestivum*	98.07	13	2	2	
	Alpha-amylase inhibitor 0.19	P01085	*Triticum aestivum*	82.99	33	2	2	
	Chymotrypsin inhibitor WCI	P83207	*Triticum aestivum*	58.51	25	2	2	
**100088B**	Alpha-amylase inhibitor 0.28	P01083	*Triticum aestivum*	99.18	69	10	10	
	Glutenin high molecular weight subunit DY10	P10387	*Triticum aestivum*	99.16	23	7	7	
	Glutenin high molecular weight subunit DX5	P10388	*Triticum aestivum*	99.13	12	7	7	
	Serpin-Z2B	P93692	*Triticum aestivum*	99.13	39	8	7	
	Alpha-amylase/trypsin inhibitor CM3	P17314	*Triticum aestivum*	99.09	49	8	7	
	Beta-amylase	P93594	*Triticum aestivum*	99.05	23	7	7	Multispecies identification: Hordeum vulgare
	Alpha-amylase inhibitor 0.19	P01085	*Triticum aestivum*	99.04	78	10	10	
	Alpha-amylase/trypsin inhibitor CM16	P16159	*Triticum aestivum*	98.92	55	9	9	
	Alpha-1-purothionin	P01544	*Triticum aestivum*	98.84	38	4	2	
	Alpha/beta-gliadin clone PW1215	P04726	*Triticum aestivum*	98.78	30	5	2	
	Alpha-amylase/trypsin inhibitor CM1	P16850	*Triticum aestivum*	98.75	39	5	2	
	Puroindoline-A	P33432	*Triticum aestivum*	98.73	45	7	7	
	Alpha-amylase/trypsin inhibitor CM2	P16851	*Triticum aestivum*	98.38	39	4	2	
	Purothionin A-1	P01543	*Triticum aestivum*	98.38	24	6	3	
	Aspartate aminotransferase, cytoplasmic	M7YLN2	*Triticum urartu*	98.35	13	3	3	Multispecies identification: Oryza sativa
	Gamma-gliadin B	P06659	*Triticum aestivum*	98.34	22	5	5	
	Avenin-like b3	P0CZ06	*Triticum aestivum*	98.30	16	4	2	
	Avenin-like b1	Q2A783	*Triticum aestivum*	98.30	16	4	2	
	Type-5 thionin	Q05806	*Triticum aestivum*	98.11	37	3	3	
	Chitin-binding type-1 domain-containing protein	A0A446YZN0	*Triticum durum*	97.96	25	3	2	Multispecies identification: Hordeum vulgare; Secale cereale
	Protein disulfide-isomerase	P52589	*Triticum aestivum*	95.93	8	3	3	Multispecies identification: Hordeum vulgare
	Puroindoline-B	Q10464	*Triticum aestivum*	94.84	32	6	6	
	Chymotrypsin inhibitor WCI	P83207	*Triticum aestivum*	92.04	25	2	2	
	Non-specific lipid-transfer protein	P24296	*Triticum aestivum*	88.01	41	2	2	
	Non-specific lipid-transfer protein 2G	P82900	*Triticum aestivum*	84.47	51	5	2	
	Trypsin/alpha-amylase inhibitor CMX1/CMX3	Q43723	*Triticum aestivum*	84.37	22	3	3	
	Triosephosphate isomerase cytosolic	P34937	*Hordeum vulgare*	84.01	10	2	2	Multispecies identification: Secale cereale
	Alpha/beta-gliadin A-V	P04725	*Triticum aestivum*	83.87	14	4	2	
	Wheatwin-1	O64392	*Triticum aestivum*	61.68	11	2	2	
	16.9 kDa class I heat shock protein 1	P12810	*Triticum aestivum*	61.68	13	2	2	
	Protein H2A.7	Q43312	*Triticum aestivum*	61.66	25	2	2	Multispecies identification: Oryza sativa; Mytilus edulis; etc.
	Carbonic anhydrase chloroplastic	P40880	*Hordeum vulgare*	60.88	9	2	2	
**100088D**	Alpha-amylase inhibitor 0.28	P01083	*Triticum aestivum*	98.21	27	3	3	
	Alpha-amylase inhibitor 0.19	P01085	*Triticum aestivum*	79.95	37	3	3	
	Purothionin A-1	P01543	*Triticum aestivum*	98.88	29	9	5	
	Alpha-1-purothionin	P01544	*Triticum aestivum*	98.84	40	7	4	
	Glutenin high molecular weight subunit DY10	P10387	*Triticum aestivum*	98.74	7	4	4	
	Alpha-amylase/trypsin inhibitor CM16	P16159	*Triticum aestivum*	61.46	28	4	4	
	Alpha-amylase/trypsin inhibitor CM2	P16851	*Triticum aestivum*	83.47	52	6	6	
	Alpha-amylase/trypsin inhibitor CM3	P17314	*Triticum aestivum*	99.05	48	7	6	
	Non-specific lipid-transfer protein	P24296	*Triticum aestivum*	97.25	80	8	8	
	Alpha-2-purothionin	P32032	*Triticum aestivum*	98.86	40	9	5	
	Puroindoline-A	P33432	*Triticum aestivum*	99.00	55	9	9	
	Protein disulfide-isomerase	P52589	*Triticum aestivum*	97.82	8	4	4	
	Non-specific lipid-transfer protein 2G	P82900	*Triticum aestivum*	98.39	65	7	5	
	Beta-amylase	P93594	*Triticum aestivum*	98.10	8	4	4	Multispecies identification: Horedum vulgare
	Chymotrypsin inhibitor WCI	P83207	*Triticum aestivum*	91.58	39	4	4	
	Puroindoline-B	Q10464	*Triticum aestivum*	61.23	9	2	2	

Legend of each column:Sample: Number classification of the archeological pottery; Proteins: The protein's header information; UniProt Id: The UniProt Accession Number as reported in the database; Taxa: Organismal origin. Proteins from different species that are supported by a common set of peptides and could not be differentiated based on MS/MS analysis alone, are also reported as multispecies identification; Protein InChorus Score: The protein confidence score calculated as percentage score when the protein was identified by both PEAKS and Mascot engines (InChorus tool). The InChorus protein percentage score is calculated as the weighted sum of the protein's supporting peptides. The PeptideProphetTM method (Keller et al. Anal. Chem. 2002, 74:5383–92) is then applied to the weighted sum scores of all proteins to convert to a probability score. Coverage: The percentage of the protein sequence that is covered by supporting peptides. #Peptides: The number of high-confidence supporting peptides. #Unique: The number of high-confidence supporting peptides that are mapped to only one protein. Unique peptides with the same sequence but different modifications are only counted once in this number

During the Peet excavation in 1909, the materials retrieved were put in large wooden boxes without being previously cleaned or subject to any treatment. After 1958, those same boxes were placed in the store-room of the National Museum of Archaeology of Valletta. No record of any study or treatment of the pottery between 1909 and 1958, and 1958 and 2017 exists in the archive of the Museum. Before sampling, dust was removed from the ceramic fragments using a brush with soft bristle. Encrustations present on the surface of all the samples here investigated were not removed. Nitrile gloves were used during this process and labeled Ziploc bags were employed to store the samples.

### Sample preparation for proteomics analyses

The improvement in resolution and accuracy of analytical techniques has required a parallel enhancement in sample preparation procedures to avoid contamination with extraneous species or other molecules that could alter the correct interpretation of the data. Therefore, to minimize contamination, protein extraction and sample handling were performed in a laboratory “clean room” dedicated to ancient protein analysis and using dedicated chemicals, lab glassware, and equipment. Surfaces and equipment were washed with 50% 2-propanol before the use. Non-latex gloves were used.

The nine samples were initially weighed individually and where possible, from 0.1 to 0.5 g were taken. Some samples, which already had sufficient weight and were statistically significant (each piece has an entire section from the inner surface to the outer surface of the ceramic sample), were not further sampled. Others were cut with a micrometric saw with a nano-diamond surface coating (Struers Minitom) at minimum speed to avoid increasing the sample temperature and altering any residues that may be present. After being weighed, each sample was inserted in a glove box with nitrogen gas flow and was ground using an agate mortar and pestle until an impalpable powder was obtained. The samples were weighed one last time to ensure that the amount of powder was sufficient for the analyses and inserted in 1.5 mL Eppendorf. All the equipment was rigorously cleaned with 2-propanol and MilliQ® water between the preparation of one sample and the next. Protein extraction was performed using modified protocols previously reported (Dallongeville et al. 2011; Greco et al. 2018). In particular, to maximize the protein extraction, the powder of each sample (between 30 and 200 mg) was divided into two aliquots and processed as follows:

- the first aliquot was treated in acidic conditions, pH = 2 (0.1% of trifluoroacetic acid, TFA, and 4% Sodium-dodecyl-sulfate solution, SDS) for 30 min at 95 °C;
- the second one was used for the extraction of protein content in alkaline reducing conditions, pH = 8.5 (4% SDS, Tris/HCl 100 mM, and 0.1 M dithiothreitol, DTT) for 30 min at 95 °C.

The volume of the extraction solution used was 5 times (in µL) the amount of sample (in mg). Protein extracts were desalted and purified from non-protein contaminants using the PlusOne 2-D Clean-Up kit (GE Healthcare Life Sciences) according to the recommendations of the manufacturer (https://www.gelifesciences.com/en/ca). The obtained pellets were dissolved in 100 µL of 50 mM ammonium bicarbonate (pH 8.3) and DTT 0.1 M to reduce the proteins (3 h, room temperature). The protein concentration for each extract was determined by a fluorimetric assay using the Qubit Protein Assay kit with the Qubit 1.0 Fluorometer (ThermoFisher Scientific, Milan, Italy) (Saletti et al. 2018). Finally, samples were alkylated with 0,2 M IAA (1 h, in the dark at 20 °C) and digested by porcine trypsin (Sequencing Grade Modified Trypsin, Porcine, lyophilized, Promega) at an enzyme–substrate ratio of 1:50 (overnight, 37 °C). The resulting peptide mixture solutions were dried under vacuum (Concentrator Plus, Eppendorf), re-dissolved in 50 µL of 5% aqueous formic acid (FA), filtered by ultracentrifugation (750 µL, 0.2 µm Nonsterile Micro-Centrifugal Filters, Sepachrom, Rho, Milan), and analyzed by UHPLC/high-resolution nanoESI–MS/MS.

Two modern control samples (bovine milk and wheat flour) were also prepared (see Supporting Material). Modern control samples were processed and analyzed by proteomics on the same way of archeological samples.

### Mass spectrometry analysis

Mass spectrometry data were acquired via a Thermo Fisher Scientific Orbitrap Fusion Tribrid® (Q-OT-qIT) mass spectrometer (Thermo Fisher Scientific, Bremen, Germany). Liquid chromatography was carried out using a Thermo Scientific Dionex UltiMate 3000 RSLCnano system (Sunnyvale, CA). One microliter of peptide mixture was loaded onto an Acclaim ®Nano Trap C18 Column (100 µm i.d. × 2 cm, 5 µm particle size, 100 Å). After washing the trapping column with solvent A (H_2_O + 0.1% FA) for 3 min at a flow rate of 7 μL/min, the peptides were eluted from the trapping column onto a PepMap® RSLC C18 EASY-Spray column (75 µm i.d. × 50 cm, 2 µm particle size, 100 Å) and separated by elution at a flow rate of 0.25 µL/min at 40 °C by a linear gradient of solvent B (ACN + 0.1% FA) in A, 5% for 3 min, followed by 5% to 65% in 85 min, 65% to 95% in 5 min, finishing by holding 95% B 5 min, 95% to 5% in 10 min and re-equilibrating at 5% B for 15 min. The Acclaim ®Nano Trap C18 and PepMap® RSLC C18 EASY-Spray columns were dedicated to this set of samples and not previously used. The eluting peptide cations were converted to gas-phase ions by electrospray ionization using a source voltage of 1.75 kV and introduced into the mass spectrometer through a heated ion transfer tube (275 °C). Survey scans of peptide precursors from 200 to 1600 m/*z* were performed at 120 K resolution (@ 200 m/*z*). Tandem MS was performed by isolation at 1.6 Th with the quadrupole, HCD fragmentation with a normalized collision energy of 35, and rapid scan MS analysis in the ion trap (low-resolution MS/MS analysis). Only those precursors with charge state 2 ÷ 4 and intensity above the threshold of 1∙10^3^ were sampled for MS^2^. The dynamic exclusion duration was set to 60 s with a 10 ppm tolerance around the selected precursor and its isotopes. Monoisotopic precursor selection was turned on. The instrument was run in full speed mode with 3 s cycles, meaning it would continuously perform MS^2^ events until the list of non-excluded precursors diminished to zero or 3 s, whichever is shorter. MS/MS spectral quality was enhanced by enabling the parallelizable time option (i.e., using all parallelizable time during full scan detection for MS/MS precursor injection and detection). Mass spectrometer calibration was performed by using the Pierce® LTQ Velos ESI Positive Ion Calibration Solution (Thermo Fisher Scientific). MS data acquisition was carried out by utilizing the *Xcalibur* v. 3.0.63 software (Thermo Fisher Scientific). Moreover, a blank laboratory extraction was carried out and analyzed under the same experimental conditions used for ancient samples. Only a meager number of background proteins was identified by database search in blank laboratory extract (see Supplementary Material).

To avoid cross-contamination with other biological samples, all solvents were prepared freshly and ancient samples were not processed or analyzed in one batch with modern references. To avoid carryover during nLC–MS/MS runs, from three to five blank runs were performed before each analysis using the same gradient program. Spectra acquired in the last blank run were searched by PEAKS software against the SwissProt database without species origin restrictions and using the same parameters of the archeological samples (see next paragraph).

### Database search and protein identification by PEAKS software

nLC–MS/MS data were analyzed and searched against the comprehensive (all species) SwissProt protein sequences database (January 2019 release, containing 558,475 entries) using integrated PEAKS De Novo sequencing software (v. 10.0, Bioinformatics Solutions Inc., Waterloo, ON Canada) and the Mascot algorithm (Matrix Science, London, UK, version 2.5.1). The first step of database search was carried out using the following parameters: (1) semi-tryptic peptides with a maximum of 3 missed cleavage sites; (2) cysteine carbamidomethylation as a fixed modification; (3) oxidation of methionine, the transformation of N-terminal glutamine and N-terminal glutamic acid residue to pyroglutamic acid form, and the deamidation of asparagine and glutamine as variable modifications. Particularly, the semi-tryptic option was chosen because it is well known that during the hydrolysis of proteins with trypsin, not only specific peptides (i.e., peptides resulting from hydrolysis at the carboxylic side of lysine and arginine) are formed, but also aspecific peptides (i.e., resulting from aspecific cleavages) have commonly been annotated. For instance, during hydrolysis by trypsin the aspecific cleavages after Tyr, Trp and Phe are the most observed (Burkhart et al. 2012; Perutka et al. 2018). On the other hand, the occurrence of spontaneous non-enzymatic peptide backbone cleavage is expected as the degradation effect in ancient samples. Then, based on previous observation of ancient proteome degradation (Cappellini et al. 2012), the unassigned “de novo only” spectra were examined by an additional search function of PEAKS, the Multi-Round Search, which allowed to examine the possible presence of post-translational modifications (unchecked in the first round of search) such as hydroxylation of proline, kynurenine formation, lysine, and arginine carbonylation.

The precursor mass tolerance threshold was set to 15 ppm and the maximum fragment mass error was set to 0.6 Da. Peptide spectral matches (PSM) were validated using a Target Decoy PSM Validator node based on q-values at a 1% False Discovery Rate (FDR). The Mascot score and PEAKS score thresholds for Peptide spectral matches (PSMs) were set to obtain for each database search FDR values, for PSMs, Peptide sequences, and Proteins identified, below the 1.0% value. Finally, all the protein hits obtained by these two approaches were processed by using the *inChorus* function of PEAKS. This tool combines the database search results of PEAKS software with those obtained by the Mascot search engine with the aim not only to increase the coverage but also the confidence since the engines use independent algorithms. Each peptide identified is reported with a percentage confidence score (*InChorus* score), reflecting the probability that the peptide–spectrum match is correct. The percentage score is calculated by the empirical calculation used in PeptideProphet™ (Keller et al. 2002). The protein score is also reported as a percentage score, calculated by adding the peptides score to identify a given protein up to a weighted sum. The PeptideProphet™ method is then applied to the weighted sum scores of all proteins to convert to a probability score. Only protein hits with a minimum of *InChorus* protein score of 50%, sequence coverage above 5% and at least two unique peptides, both having an individual *InChorus* score above the threshold of 50%, were considered valid. Proteins belonging to the same species identified by the same peptides and that could not be differentiated based on MS/MS analysis alone were grouped to satisfy parsimony principles (groups of parsimony). Proteins from different species identified by the same peptides were also grouped to satisfy parsimony principles and reported as multispecies identification (see Supplementary Tables).

The mass spectrometry proteomics data have been deposited to the ProteomeXchange Consortium (http://proteomecentral.proteomexchange.org) via the PRIDE partner repository (Vizcaino et al. 2013) with the dataset identifier < PXD022848 > .

### Calculation of deamidation level by MaxQuant software

MS raw data were also processed by MaxQuant (MQ) software 1.6.3.4 ( https://www.maxquant.org/) to further investigate about deamidation level of the proteins identified (Cox et al. 2008).

In this search, a more specific database from Uniprot (Swiss-Prot and TrEMBL), which included the proteins identified in the PEAKS search, was used. A database including all the milk proteins from the *Bovidae* family and a database including all the proteins from *Triticum* was used for the potentially milk-containing samples (100041 M, 100051A, and 100051D) and wheat-containing samples, respectively (100087C, 100087D, 100088B, and 100088D). Moreover, the common Repository of Adventitious Proteins (c-RAP) contaminant database was enabled in the database search.

Database search was carried out using the following parameters: i) full tryptic peptides with a maximum of 3 missed cleavage sites; ii) cysteine carbamidomethylation as a fixed modification; iii) oxidation of methionine, the transformation of N-terminal glutamine and N-terminal glutamic acid residue to pyroglutamic acid form as variable modifications, hydroxyproline oxidation, and the deamidation of asparagine and glutamine as variable modifications. Match type was “match from and to”. The decoy mode was “revert”. PSM, Protein and Site decoy fraction FDR were set at 0.01 as threshold for peptide and protein identifications. Minimum score for modified and unmodified peptides was set at 40. All the other parameters were set as default. The principles of parsimony were still applied.

An estimation of the percentage of deamidation for each sample was calculated with the aid of a freely available command-line script for Python 2.x (https://github.com/dblyon/deamidation), which use the MaxQuant “evidence.txt” file. The calculations were done separately for potentially original peptides and potential contaminants peptides as previously reported in Mackie et al. 2018. Briefly, for each peptide containing N and Q residues, the ratio between the number of the residues in the modified form and the total number of the residues was calculated and this value was then multiplied for the intensity of the peptide. The values obtained were summed. The result was then divided by the total sum of all intensity values of the peptides in the modified and unmodified form. 1000 bootstrap iterations were applied to calculate mean, standard deviation, and 95% lower and upper confidence intervals and estimate the calculation error (see the Supplementary Material for details).

## Results

Protein extracts from nine powdered samples were analyzed by a MS-based proteomic approach. A preliminary hypothesis about the use of these ancient pottery and the food contained inside was formulated based on archeological studies, and comparisons offered by the contemporaneous Sicilian and South-Italian repertoire of shapes (Recchia et al. 2000). The cups with inverted rims (samples 100041 M and 100061B), usually pedestalled, are considered as sort of serving dishes for semi-solid food more than drinking vessels, while for the strainer vessels there are different hypotheses based on their typological variations. The cylindrical strainer (100051A) has been traditionally interpreted, without any solid evidence though, as connected with cheese making due only to its resemblance to typical wicker cheese baskets of the rural culture. The strainer example (100051D), has instead been identified as a portion of a strainer spouted jug, a typical Sicilian, Aegean, and Levantine Late Bronze and Early Iron Age vessels, considered as a container of a beer-like fermented beverage (Leighton et al. 1981). The five examples of large storage jars (100061H, 100087C, 100087D, 100088B, and 100088D) are commonly interpreted as multi-purpose large size containers of liquids (such as olive oil and wine) or cereals (Tanasi 2020). At times, contents of intact examples can be suggested by the presence of a spout set by the base, used to empty and clean those filled with liquids, as it happens in culturally and chronologically compatible contexts in Sicily. In those specific cases, storage jars appear not to have been subject to reuse or change in the type of content (Albanese Procelli et al. 2003). In the case of these five jars, no clear clues about their function could have been obtained due to their fragmentary conditions. Proteomic data of these nine samples identified hundreds of peptides. Most of them were from keratins and human skin-derived proteins, but many food proteins were also recognized. Taking into account that archeological materials have intrinsic contamination problems that can be originated at nearly any stage of burial, excavation, storage, and analysis, authentication and validation criteria are needed in order to discriminate ancient endogenous proteins from contaminants. First of all, it is important to highlight that, unlike their modern counterparts, the chemical structure of ancient proteins is altered by a series of complex diagenetic reactions (Cleland et al. 2020). Even if this is still an open question, the deamidation level of glutamine and asparagine is one of the proposed biomolecular indicators of deterioration and natural aging of proteins in artistic and archeological materials (Schroeter et al. 2016). It is important to note that asparagine and glutamine deamidate at vastly different rates, with glutamine being considerably slower (Robinson et al. 2001a; b). Consequently, glutamine deamidation has become more widely used as a means to assess time-dependent damage in ancient sample age. In particular, recent studies on paleontological bone have observed that known contaminants present in ancient protein extracts show very little to no deamidation, while endogenous bone collagens and non-collagenous proteins generally display medium or extensive deamidation (Welker et al. 2016, 2017). However, many environmental conditions, including pH, temperature, protein sequence, protein structure, ionic strength, etc., can affect the rate and total deamidation levels observed in a sample. Therefore, the only use of this modification as a definitive marker to estimate the absolute age of proteins derived from archeological materials may lead to the incorrect classification of exceptionally well-preserved, incompletely deamidated ancient sequences as contamination (Schroeter et al. 2016). Indeed, ancient proteins may also undergo other age-dependent oxidative damages (e.g., kynurenine formation, methionine or tryptophan dioxidation, carbonylation of lysine and arginine etc.) as observed in artificially aged wool and mammoth bone from permafrost (Cappellini et al. 2012) or experience spontaneous peptide backbone cleavages, which should be also examined. Concerning the last damage, protein fragmentation frequently occurs due to spontaneous non-enzymatic peptide backbone cleavage at the carboxyl side of asparagine, aspartic acid, glutamine, and glutamic acid (Joshi et al. 2004). On the other hand, it is also important to highlight that temperature, burial environment, and the fossil chemistry all influence how quickly a protein may decay. Moreover, it is not clear what mechanisms slow down decay, so that some protein sequences might show low damages after thousands of years. As a general result, it remains difficult to know where to look for these ancient sequences.

### Proteomic results: identification of proteins and calculation of deamidation level

The complete collection of proteomic data (i.e., list of the identified proteins, taxonomy, percentage of sequence coverage, *InChorus* score, sequence of the corresponding peptides, etc.) obtained by PEAKS software, relative to the nine samples here investigated, is reported in the Supplementary Table S1.

A total of 758 peptides were characterized. 470 were from human proteins; whereas, 288 were related to milk or cereal proteins. Results reveal that, in many samples, a very high level of human skin-derived peptides, which probably represent contaminants, was detected. In particular, these peptides allowed the identification of 97 human skin-derived proteins, 57 cereal proteins, and 7 *Bovinae* milk-derived proteins. In detail, in the samples 100061B and 100061H, only human skin proteins were identified. Samples 100041 M and 100051D present a very high level of human skin protein and only one (α_s1_-casein) and four milk-derived proteins (α_s1_-, α_s2_-, β-, and κ-casein), respectively. Instead, sample 100051A does not present human contaminants, but shows only two milk proteins, a β-casein and β-lactoglobulin, from *Bovinae* species (*Bos taurus or Bubalus bubalis*). With respect to this sample, it is interesting to note that the two β-lactoglobulin-derived peptides (TPEVDDEALEKFDK and TPEVDDEALEK) contain an important variant site that distinguishes *Bovinae* species from sheep. Indeed, an aspartic acid (D) in *Bovinae* (underlined in the previous peptides), is an asparagine (N) in sheep. Considering that the deamidation of asparagine results in its conversion to aspartic acid, protein identification software is unable to distinguish an unmodified *Bovinae* residue (D) from a deamidated sheep residue (DeN) at this position (Hendy et al. 2018a; Warriner et al. 2014). As a consequence, species discrimination is not possible at this stage. Finally, in the samples 100087C, 100087D, 100088B, and 100088D, extracted from four large storage jars (see Table 1) which were probably used for the storage of oil, wine or cereals, many cereal proteins were identified. On the contrary, only few human-derived proteins were detected. In detail, a total of nine wheat proteins (five in the sample 100087C and four in the sample 100087D) were identified; whereas, only one human protein was found (sample 100087D). Proteomic analysis of the samples 100088B and 100088D revealed the presence of 32 and 16 cereal proteins (from *Triticum* and *Hordeum* species), respectively, and a total of 14 human proteins (10 in the 100088B sample and 4 in the 100088D).

**Fig. 3:**
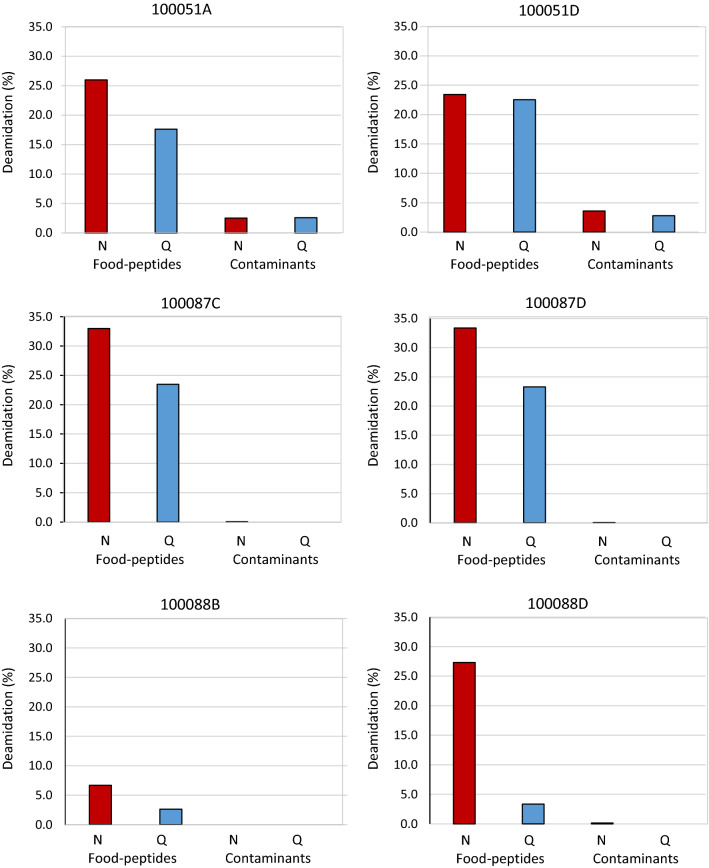
Deamidation level (reported as percentage) of asparagine (N) and glutamine (Q) amino acids in the archeological samples analyzed

To discriminate food-derived proteins, as original components of our samples, from human proteins which probably represent contaminant components related to the post-excavation history of the samples, two aspects were evaluated: peptides deriving from non-enzymatic cleavages, and the deamidation level of asparagine (Asn) and glutamine (Gln) residues. As mentioned above, peptides generated by non-enzymatic cleavages at the carboxyl side of asparagine, glutamine, aspartic acid, and glutamic acid may be related to the degradation effect in ancient samples (Joshi et al. 2004). Moreover, Asn and Gln residues naturally deamidate over time (Robinson et al. 2001a), and these modifications may be used as biomolecular indicators of deterioration and natural aging of proteins in archeological materials. Concerning the evaluation of non-enzymatic cleavages, our results (Supplementary Table S1) show that about 10% (28 out of 288) of food-derived peptides arise by non-enzymatic cleavages, whereas only 2% (9 out of 470) of the human peptides are related to non-enzymatic cleavages. Then, by MQ software, we calculated the deamidation level of asparagine and glutamine residues in food-derived peptides identified in the archeological samples and compared it with that of human-derived peptides (classified as potential contaminants) (see Supporting Information and Supplementary Table S2). With the exclusion of the sample 100041 M, in which the score of all the deamidated and unmodified peptides was below the minimum score set, in the other six samples the deamidation level has been determined. Figure 3 shows that, in general, food-derived peptides present a deamidation level which is five to seven fold as high as that of the potential contaminants (e.g., human-derived peptides). Particularly, in the samples 100051A, 100051D, 100087C, 100087D, and 100088D, the level of deamidation ranges from 23 to 33% for asparagine residues and from 3 to 23% for glutamine residues in food-derived peptides. On the contrary, for the human peptides the level of deamidation ranges from 0 to 4% asparagine residues, and from 0 to 3% for glutamine residues (Fig. 3 and Supplementary Material for details). Instead, the level of deamidation of food-derived peptides in sample 100088B is lower than that in the other samples (6.7% Asn and 2.6% Gln). The lower level of deamidation observed for this sample might be probably due to well-preserved endogenous ancient components. On the other hand, unless other complementary chemical analyses, it cannot be excluded a priori that also food-derived peptides detected in this sample are instead related to the post-excavation history of the samples.

Taking into account that deamidation may also occur in vitro during proteomics sample handling (Xing et al. 2008), we also determined the deamidation level in two modern control samples: a commercial fresh milk sample and a wheat flour sample (see Supplementary Material for details). On average, 8.0% Asn and 9.1% Gln residues in milk, and 7.4% Asn and 4.8% Gln residues in wheat control sample resulted deamidated (Table S3 and Figure S1).

## Discussion

Paleoproteomics is currently a growing research area that, by the use of liquid chromatography–tandem mass spectrometry (LC–MS/MS)-based approaches (known as “shotgun” methods), may offer an alternative strategy for identifying foodstuffs prepared in ceramics (Craig et al. 2000). It should be highlighted that, even if “shotgun” proteomic approaches permit the identification of a wide range of proteins in a sample, their application to archeological artifacts has so far been limited to exceptionally well-preserved examples from waterlogged, cold or arid contexts (Buckley et al. 2013; Solazzo et al. 2008).

In this work, we applied a shotgun proteomic approach to proteins extracted from ceramics of the pottery of the Maltese site of Il-Qlejgħa tal-Baħrija, the guide-site for the local Iron Age (11th–eighth century BCE), intending to confirm the preliminary archeological hypothesis about the presence of cereal storage practices, which could offer support to the hypothesis of an emergent proto-urban society based on economic accumulation redistribution of the surplus, and eventually to shed light on indigenous foodways. Our data also highlighted the impact of one of the many difficulties in identifying ancient proteins: the cross-contamination from modern proteins. Protein contamination of ancient and archeological materials are mainly, but not only, due to keratins and human skin-related proteins, that can occur at nearly any stage of the interaction of human agents with the artifact (use, burial, excavation, storage, and sample extraction) and may potentially provide false insights into protein composition, phylogeny, and protein modification, and may also affect the detection and identification of the ancient proteins. Consequently, in compliance with protection guidance for archeological samples, some precautions were adopted to minimize the effects of contamination from modern proteins (see material and methods section).

Proteomics data obtained for many samples here investigated revealed a high level or the exclusive presence of human skin protein contaminants and low amounts of food-derived proteins. To facilitate the interpretation, we subdivided the identified proteins into two groups: human-derived proteins, considered as a common background, were grouped together and classified as potential contaminants; the remaining protein hits derived from foods constituted the second group and were considered as potential authentic ancient proteins. Then, to distinguish between potential contaminants and authentic ancient proteins, we examined two aspects that may be related to diagenetic effects in ancient samples: the number of peptides generated by non-enzymatic cleavages and the deamidation level of Asn and Gln residues. As a general trend, we observed that the number of food-peptides arising by non-enzymatic cleavages is about five fold as high as that of potential contaminants. Similarly, food-derived peptides show a much higher deamidation level regarding that of human contaminants, which is the same as the modern samples used as control. Altogether these data seem to indicate that food-derived proteins identified in our samples can be considered as authentic components. On this respect, interesting archeological implications have the identification of milk in samples 100051A and 100051D. While such discovery for sample 100051A might confirm the common interpretation of such shape as related to cheese making, it is very remarkable that it is instead the case of sample 100051D. This tableware, traditionally interpreted as a beer-like fermented beverage container of foreign origin, eventually in Malta could have been used in a completely original and different way. In light of these archeological implications, it is important to note that the identification of milk proteins as authentic ancient components, and not contaminants, should be confirmed by complementary chemical analyses.

The presence of cereals-derived proteins in samples 100087C, 100087D, 100088B, and 100088D may inform us of the function of the four large storage jars in question as containers of cereals. On the other hand, complementary tests (e.g., lipid biomarker analysis) (Hammann et al., 2018) should be carried out to dispel or not hypothesize that these four jars could have also been contained olive oil or wine. The lower level of deamidation (the same of that calculated for modern control samples) observed for the food-derived peptides identified in the sample 100088B poses the question of whether these peptides may be considered well-preserved endogenous ancient components or are related to modern contaminants. Therefore, to validate the authenticity of these proteomics identification complementary chemical analyses should be performed. At this stage, it can only be hypothesized that encrustations, a layer rich of mineral-organic binder, retained on the surface of all the pottery here investigated, may have facilitated a long-term archeological preservation of ancient proteins and peptides, protecting from degradation and damage driven by external factors.

Finally, the lack of archeozoological and paleobotanical data for the settlement at Il-Qlejgħa tal-Baħrija makes the proteomic analysis outcomes even more relevant in terms of contributing to the reconstruction of foodways and dietary habits of that ancient community. In this respect, the assignment of many cereal proteins to *Triticum* species appears interesting. Indeed, the paleobotanical data, available for other sites in the Maltese archipelago, offer an interesting trend in terms of growth and consumption of cereals (Fiorentino et al. 2013). For most of the Neolithic and Copper Age (5000–2000 BCE ca.), *Hordeum* appears to be predominant with few instances identified of *Triticum*. Subsequently, in the Early Bronze Age (2000–1500 BCE ca.) barley decreases gradually in favor of naked kinds of wheat which reached their peak at this end of this period. But in the second half of the 2nd millennium BCE (*facies* of Borġ in-Nadur—1500–750 BCE ca.) barley increases significantly, up to 55% of the sample assemblage. At least for the specific case of the settlement at Il-Qlejgħa tal-Baħrija, our data, even if obtained by a small selection of vessels, instead seem to indicate that wheat was still consumed.

## Conclusions

Food habits are constructed by a broad range of cultural, ideological, and interpersonal factors such as status, religion, gender, age, wealth, and more. In this perspective, food is not just biologically necessary, but also it becomes a cognitively prominent “material culture” that plays an active role in constructing and negotiating social distinctions. While such very peculiar material in the case of classical antiquity is mainly addressed through the study of written and iconographic sources, concerning prehistoric civilizations, chemical techniques of investigation are currently the sole approach available. In particular, archeological ceramics occupy a place of absolute importance among the artifacts that have come down to us since prehistoric times, taking with them hidden biomolecular information about their function.

Traditional hypotheses on the contents of ancient vessels are based on comparisons with later examples, for which there is some historical certainty, or simply on the observation of shape and decorative style peculiarities. In this perspective, paleoproteomic study of ancient residual food-related compounds absorbed by pottery represents a powerful alternative approach that may inform us about dietary habits, culinary practices, and foodways. Moreover, it can also provide the opportunity for improved tissue and taxonomic resolution, as the identification of proteotypic peptides, i.e., peptides that are unique to the protein sequence specific for an individual organism and not in common to other species, permits to discriminate the organism of origin of the identified proteins. Concerning the set of samples here investigated, even if limited to a small selection of vessels, proteomics results revealed that wheat was central in the diet of the ancient community of Il-Qlejgħa tal-Baħrija. However, it is also important to note that in paleoproteomic studies protein identification is obtained investigating database which contain the nearly complete proteomes of many model organisms (e.g., *Mus musculus*, *Homo sapiens,* etc.), but comprise only partial proteomes of other taxa (e.g., *Capra hircus*, *Ovis aries,* etc.), included the *Triticum* species and other monocots.

In conclusion, it is important to note that as a young discipline, paleoproteomics, especially when applied to ancient ceramics, still needs to be developed in terms of efficiency of protocols and appropriate databases applied. Further studies about the PTMs characterization, amino acid composition, chemical, and physical properties of the identified peptides will give us interesting information about what determines the characteristics and stability of proteins during the time. Also, major understanding of how proteins are transferred from food to the ceramic and how they survive into the pots is needed. The mechanisms behind the preservation of ancient protein are not well understood, but are thought to involve a range of circumstances, including the composition of specific proteins (e.g., hydrophobicity properties), dry conditions, cold temperatures, encapsulation within mineral matrices, and chemical modifications. It is well known that protein residues are subject to transformations resulting from exposure to the environment. Although these modifications introduce a challenge for protein identification and authentication, they are also thought to promote preservation by rendering protein residues less susceptible to microbial attack (Barker et al. 2012, 2018).

Our results also confirm that archeological deposits are usually composed of degraded, mixed and contaminated compounds (mainly human-derived proteins), and their analysis requires a complex analytical approach, including methods to discriminate authentic from contaminant proteins. The lower deamidation level observed in a sample (e.g., 100088B) for cereal-derived peptides might be probably related, but not limited, to the presence of encrustation layer which may have well-preserved protein molecules present into the ceramic. In this respect, although the chemical and mechanical removal of encrustations in ceramic vessels is still considered good practices in archeology (Casaletto et al. 2008), these results confirm the importance of these in situ deposits for preserving protein molecules as already reported (Demarchi et al. 2016; Hendy et al. 2018b). However, it is essential to highlight that different environmental factors and the inherent properties of proteins, including their primary sequences and three-dimensional structures, might have also affected deamidation and other post-translational modifications. On the other hand, this study confirms the challenges related to the investigation of ancient proteins, especially if extracted from ceramic substrates. In this specific case, proteomic data appears attractive in terms of contributing to the reconstruction of dietary habits of the ancient community of the Maltese site of Baħrija, but it would be desirable for them to be cross-validated, in the future, by complementary analyses, such as investigation of lipid biomarkers, aDNA analysis, and carbohydrates characterization. Indeed, it is important to highlight that combining the results of ancient biomolecules (proteins, lipids, carbohydrates, and aDNA) can provide a more coherent approach to tackling complex biological questions than the use of any of these biomolecular proxies alone.

## Electronic supplementary material

Below is the link to the electronic supplementary material.

Supplementary file1 (DOCX 72 kb)

Supplementary file2 (XLSX 102 kb)

Supplementary file3 (XLSX 396 kb)

Supplementary file3 (XLSX 310 kb)

Supplementary file3 (XLSX 873 kb)
