# Supplementary material for: Paleoproteomic profiling of organic residues on prehistoric pottery from Malta
Source: Amino Acids. 2021 Feb 13;53(2):295–312. doi: 10.1007/s00726-021-02946-4 (PMC7910365; doi:10.1007/s00726-021-02946-4)
Supplement: Supplementary file 1 — Supplementary file1 (DOCX 72 kb) [file 726_2021_2946_MOESM1_ESM.docx]

##

**Paleoproteomic profiling of organic residues on prehistoric pottery from Malta**

Davide Tanasi,^1^ Annamaria Cucina,^2^ Vincenzo Cunsolo,^2,*^ Rosaria Saletti,^2^ Antonella Di Francesco,^2^ Enrico Greco,^3^ and Salvatore Foti^2^

^1^ Department of History, University of South Florida, SOC107 4202 E. Fowler Ave, Tampa, FL 33620, United States

^2^ Laboratory of Organic Mass Spectrometry, Department of Chemical Sciences, University of Catania, Viale A. Doria 6, 95125, Catania, Italy

^3^ Aix-Marseille Université, Institut de Chimie Radicalaire Service 512, AVE Escadrille Normandie Niemen - 13013 Marseille, France

Keywords: Paleoproteomics; cereal proteins; high-resolution mass spectrometry; Orbitrap Fusion Tribrid; Deamidation

* Corresponding Author:

Vincenzo Cunsolo,

Department of Chemical Sciences, University of Catania, Viale A. Doria 6, 95125 Catania, Italy.

Tel. 0039 095 7385029

Fax 0039 095 580138

E-mail: [vcunsolo@unict.it](mailto:vcunsolo@unict.it)

*Control blank sample*

A blank laboratory extraction (e.g. pieces of an unglazed terracotta pot immerged in water) was carried out and analyzed under the same experimental conditions used for ancient samples. Database search by PEAKS software identified the following list of peptides and proteins (FDR 1%)

*List of Proteins identified in blank extraction*

| **Acc. No.** | **-10lgP** | **Cover. (%)** | **#Peptides** | **#Unique** | **PTM** | **Av. Mass** | **Description** |
| --- | --- | --- | --- | --- | --- | --- | --- |
| P00698 | 67.65 | 13 | 2 | 2 |  | 16239 | Lysozyme C OS=Gallus gallus |
| P00761 | 52.71 | 9 | 1 | 1 |  | 24409 | Trypsin OS=Sus scrofa |
| C6KTD2 | 47.75 | 0 | 1 | 1 |  | 796066 | Putative histone-lysine N-methyltransferase 1 OS=Plasmodium falciparum |
| Q8TY21 | 40.81 | 6 | 1 | 1 | CM-Cys | 37112 | GTP cyclohydrolase MptA OS=Methanopyrus kandleri |
| Q27171 | 37.00 | 1 | 1 | 1 | CM-Cys | 528640 | Dynein heavy chain cytoplasmic OS=Paramecium tetraurelia |

*List of peptides identified in blank extraction*

| **Protein Acc. No.** | **Peptide** | **Unique** | **-10lgP** | **Mass** | **m/z** | **Length** | **ppm** | **z** | **RT** | **Scan** | **Start** | **End** | **PTM** |
| --- | --- | --- | --- | --- | --- | --- | --- | --- | --- | --- | --- | --- | --- |
| P00698 | K.FESNFNTQATNR.N | Y | 48.63 | 1427.6430 | 714.8287 | 12 | 0.0 | 2 | 74.39 | 12977 | 52 | 63 |  |
| P00698 | R.HGLDNYR.G | Y | 38.03 | 873.4093 | 437.7124 | 7 | 1.0 | 2 | 78.48 | 15056 | 33 | 39 |  |
| P00761 | R.LGEHNIDVLEGNEQFINAAK.I | Y | 52.71 | 2210.0970 | 737.7076 | 20 | 2.0 | 3 | 78.48 | 15073 | 58 | 77 |  |
| C6KTD2 | K.KPLLYNR.S | Y | 47.75 | 902.5338 | 452.2779 | 7 | 8.2 | 2 | 54.60 | 8146 | 4039 | 4045 |  |
| Q8TY21 | R.KMVGAEVVGLTAC(+57.02)PC(+57.02)ALEMM(+15.99)R.E | Y | 40.81 | 2338.0980 | 1170.0620 | 21 | 5.4 | 2 | 76.73 | 14172 | 152 | 172 | CM-Cys |
| Q27171 | K.C(+57.02)FLTLTQALHLRMGGSPFGPAGTGK.T | Y | 37.00 | 2616.3300 | 1309.1840 | 25 | 8.9 | 2 | 116.46 | 22125 | 1817 | 1841 | CM-Cys |

## *Preparation of modern control samples*

Once the main proteins components of the samples 100041M, 100051A, 100051D, 100087C, 100087D, 100088B and 100088D were identified through the PEAKS data analysis, modern control samples similar to the identified proteins were prepared. In particular, two type of control samples, respectively of bovine milk and wheat flour, were prepared and their deamidation rate was compared with the samples deamidation rates in order to support or reject the hypothesis of authenticity.

## *Modern control sample n.1: milk*

Milk control sample was prepared performing a simulated interaction with pottery. Pieces of an unglazed terracotta pot were sampled and immerged in full-cream milk. The samples were left in milk for four weeks. Proteins from milk control sample were, processed and analyzed by proteomics on the same way of archeologic samples, as reported in the main text.

Database search by PEAKS software allowed the identification of fifty *Bos taurus* proteins and various *bacteria* proteins (e.g. *Lactobacillus casei* and *Lactobacillus paracasei*). Milk proteins identified were four caseins (α_s1_, α_s2_, β e κ-caseins), the two most abundant siero-proteins (lactoglobulins and lactalbumins) and the less abundant siero-proteins (lactoferrin, lactoperoxidase, lactadherin, lactotransferrin) (See Supplementary Table S5).

## *Modern control sample n.2: wheat flour*

A modern sample of wheat flour of the Simeto genotype (*Triticum durum*) was used as cereal control. Proteins from wheat control sample were, processed and analyzed by proteomics on the same way of archeologic sample, as reported in the main text. Database search by PEAKS software allowed the identification of many wheat proteins and *bacteria* (See Supplementary Table S6).

## *Calculation of deamidation level in ancient and modern samples*

Taking into account that common protein background comprised human keratins together with several skin and saliva proteins introduced during sample handling, both database search and calculation of deamidation level were carried out using the common Repository of Adventitious Proteins (c-RAP) database (URL at <ftp://ftp.thegpm.org/fasta/cRAP> ), a predefined contaminants database for proteomics as background. Then, we calculated the deamidation level of asparagine and glutamine residues of potential contaminants peptides and compared it with that of food-derived peptides identified in the archaeological samples.

The code for the method of the calculation is freely available to the scientific community on GitHub (<https://github.com/dblyon/deamidation>). The script calculation is described as follow.

MaxQuant’s “evidence.txt” file was used to calculate separate deamidation rates for Asparagine (N) and Glutamine (Q). The fraction of num_N (number of Asparagines) to num_N-2-D (number of deamidated Asparagines) and the fraction num_Q (number of Glutamines) to num_N-2-D (number of deamidated Glutamines) were calculated for each peptide-to-spectrum match (PSM). The values obtained were termed respectively ratio_N-2-D and ratio_Q-2-E. The ratio_N-2-D or ratio_Q-2-E was multiplied for the “Intensity” of the PTM, the values were summed and the result divided by the total sum of all intensity values of the respective unmodified peptide sequence, obtaining a deamidation rate between 0 and 1 for each unique peptide sequence and charge state. For each peptide was calculated an average deamidation rate for Asparagine and Glutamine. The deamidation rates were averaged per sample. The latter set of values was sampled with replacement (bootstrapped) 1000 times. The mean, the standard deviation, and the 95% confidence intervals were calculated in order to achieve an estimate of the error of the calculation.

The program generates four delimited text files as output:

- Deamidation.txt (Raw Files, deamidation for N and Q, as mean, standard deviation, 95% confidence lower and upper limit)
- Number_of_Peptides_per_RawFile.txt
- Bootstrapped_values.txt (all the deamidation percentages calculated by e.g. 1000 bootstrap iterations, which are subsequently used to calculate the mean, std, and CI for shown in "Deamidation.txt")
- Protein_deamidation.txt (deamidation on the protein level, to be used with restraint since there usually are few data to acquire meaningful results, therefore no bootstrapping is applied)

## *Deamidation results in ancient samples*

**Table S2. Results of the deamidation level calculation in archaeological samples**

| **Sample** | **Peptides** | **N_Q** | **Deamidation (%)** | **Standard Deviation (%)** | **PSM** |
| --- | --- | --- | --- | --- | --- |
| 100051A | Food-derived | N | 26.0 | 10.9 | 17 |
|  |  | Q | 17.6 | 7.9 | 27 |
|  | Potential Contaminants | N | 2.5 | 2.2 | 96 |
|  |  | Q | 2.6 | 1.8 | 85 |
| 100051D | Food-derived | N | 23.4 | 12.3 | 21 |
|  |  | Q | 22.5 | 9.4 | 34 |
|  | Potential Contaminants | N | 3.6 | 1.3 | 505 |
|  |  | Q | 2.8 | 1.1 | 563 |
| 100087C | Food-derived | N | 33.0 | 9.2 | 36 |
|  |  | Q | 23.5 | 6.2 | 64 |
|  | Potential Contaminants | N | 0.1 | 0.1 | 20 |
|  |  | Q | 0.0 | 0.0 | 11 |
| 100087D | Food-derived | N | 33.4 | 11.2 | 13 |
|  |  | Q | 23.3 | 9.4 | 37 |
|  | Potential Contaminants | N | 0.1 | 0.05 | 25 |
|  |  | Q | 0.0 | 0.0 | 31 |
| 100088B | Food-derived | N | 6.7 | 2.4 | 184 |
|  |  | Q | 2.6 | 0.9 | 362 |
|  | Potential Contaminants | N | 0.0 | 0.0 | 50 |
|  |  | Q | 0.0 | 0.0 | 52 |
| 100088D | Food-derived | N | 27.3 | 7.4 | 44 |
|  |  | Q | 3.3 | 1.8 | 116 |
|  | Potential Contaminants | N | 0.1 | 0.1 | 17 |
|  |  | Q | 0.0 | 0.0 | 15 |

## *Deamidation results in modern control samples*

For the milk-control, MaxQuant search was performed using a *Bovidae* milk proteins database. In the “evidence” file, the peptides were marked as “original” if related to milk proteins and “contaminants” in the other cases in the “Raw File” column. The deamidation rate was so calculated only for milk related peptides.

For the wheat-control, MaxQuant search was performed using a *Triticum* proteins database. In the “evidence” file, the potential contaminants peptides (keratins) were removed. The deamidation levels result comparable, with value below 10% in both samples and at both Asparagine and Glutamine residues (Figure S1).

**Table S3. Results of the deamidation level calculation in modern control samples**

| **Modern Control Sample** | **N_Q** | **Deamidation (%)** | **Standard Deviation (%)** | **PSM** |
| --- | --- | --- | --- | --- |
| Milk | N | 8.0 | 1.8 | 311 |
|  | Q | 9.1 | 1.8 | 345 |
| Wheat | N | 7.4 | 0.9 | 910 |
|  | Q | 4.8 | 0.7 | 1026 |


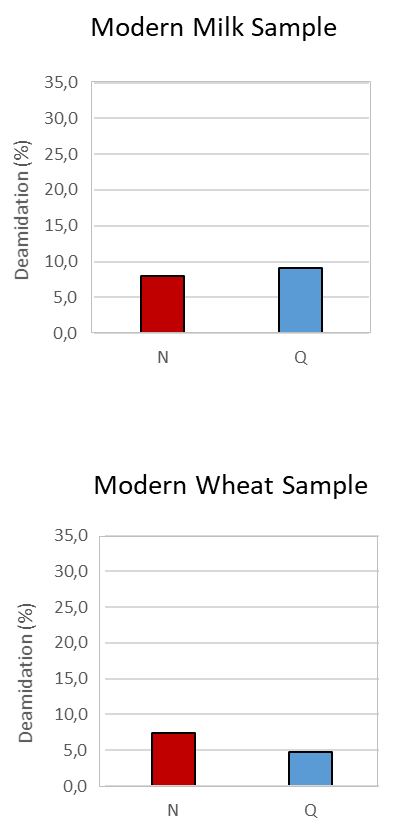


Figure S1. Average of deamidation level of asparagine (N) and glutamine (Q) amino acids in the control samples.
